# Near-simultaneous intravital microscopy of glucose uptake and mitochondrial membrane potential, key endpoints that reflect major metabolic axes in cancer

**DOI:** 10.1038/s41598-017-14226-x

**Published:** 2017-10-23

**Authors:** Caigang Zhu, Amy F. Martinez, Hannah L. Martin, Martin Li, Brian T. Crouch, David A. Carlson, Timothy A. J. Haystead, Nimmi Ramanujam

**Affiliations:** 1 Department of Biomedical Engineering, Duke University, Durham, NC 27708 USA; 2 Department of Pharmacology and Cancer Biology, Duke University, Durham, NC 27710 USA

## Abstract

While the demand for metabolic imaging has increased in recent years, simultaneous *in vivo* measurement of multiple metabolic endpoints remains challenging. Here we report on a novel technique that provides *in vivo* high-resolution simultaneous imaging of glucose uptake and mitochondrial metabolism within a dynamic tissue microenvironment. Two indicators were leveraged; 2-[N-(7-nitrobenz-2-oxa-1, 3-diazol-4-yl) amino]-2-deoxy-D-glucose (2-NBDG) reports on glucose uptake and Tetramethylrhodamine ethyl ester (TMRE) reports on mitochondrial membrane potential. Although we demonstrated that there was neither optical nor chemical crosstalk between 2-NBDG and TMRE, TMRE uptake was significantly inhibited by simultaneous injection with 2-NBDG *in vivo*. A staggered delivery scheme of the two agents (TMRE injection was followed by 2-NBDG injection after a 10-minute delay) permitted near-simultaneous *in vivo* microscopy of 2-NBDG and TMRE at the same tissue site by mitigating the interference of 2-NBDG with normal glucose usage. The staggered delivery strategy was evaluated under both normoxic and hypoxic conditions in normal tissues as well as in a murine breast cancer model. The results were consistent with those expected for independent imaging of 2-NBDG and TMRE. This optical imaging technique allows for monitoring of key metabolic endpoints with the unique benefit of repeated, non-destructive imaging within an intact microenvironment.

## Introduction

Deregulation of cellular energetics is a hallmark of cancer^1^, and metabolic profiling of tumors allows researchers to investigate the mechanisms underlying cancer progression, metastasis, and resistance to therapies^2–5^. In spite of variations in tissue site and signaling pathways, most cancers exhibit the common metabolic characteristic of increased glucose metabolism relative to normal cells^1^. The ability to perform glycolysis regardless of oxygen availability was coined the “Warburg effect”, after Otto Warburg, who first described aerobic glycolysis in cancer^6^. More recently, the Warburg effect is challenged by a growing number of studies showing that many cancers rely heavily on both mitochondrial metabolism and glycolysis to meet the increased energy demands required for progression^7–9^. Even the most glycolytic tumor types may produce only 50–60% of their ATP by glycolysis, with the balance from mitochondrial metabolism^10,11^.

Several important phenomena highlight the importance of measuring both glycolytic and mitochondrial metabolism. Tumors with increased capacity for both glycolysis and oxidative phosphorylation tend to be aggressive, with the ability to survive stressors such as cycling hypoxia or low nutrient availability. This adaptable metabolic phenotype promotes negative outcomes such as increased migration^12^, metastatic propensity^13^, and drug resistance^14^. Further, recent evidence shows that some metastatic tumors rely almost primarily on mitochondrial metabolism so that they can utilize “waste products” such as lactate from the surrounding microenvironment^15,16^; this has been coined the “Reverse Warburg effect”^15^. Metabolic changes are also essential for tumor cells that evade therapy and eventually recur. Some studies have found that dormant cells exhibit a relatively increased dependence on mitochondrial metabolism^17,18^, confirming that the ability of tumor cells to shift their metabolism between glycolysis and oxidative phosphorylation is essential for survival in changing environments^19^.

There are a number of imaging methods that enable organ-level imaging of metabolic endpoints *in vivo* with a resolution of 1–2 mm^20^. Positron Emission Tomography (PET) and Magnetic Resonance Spectral Imaging (MR(S)I) are two such technologies^20^. PET imaging is a well-accepted technique for measuring glucose uptake using fluorodeoxyglucose ([18 F]FDG) as a tracer^21^. PET can be also used to image tissue hypoxia by incorporating additional probes (e.g. [18 F]FMISO)^22^. MR(S)I can report on both mitochondrial metabolism and glycolysis endpoints^23,24^ using ^31^P or hyperpolarized ^13^C labeled compounds as tracers, and MRI can also quantify vasculature based on blood flow effects^25^.

At the cellular level, measurements of glycolysis and mitochondrial metabolism are performed most commonly with *in vitro* cellular metabolism analyzers such as the Seahorse extracellular flux analyzer (Agilent, USA)^26–31^. The Seahorse assay measures two metabolic endpoints: the extracellular acidification rate (ECAR), which reports indirectly on glycolysis, and oxygen consumption rate (OCR), which reports on oxidative phosphorylation. These assays are particularly useful in high-throughput experiments and can be used to compare the ratio of glycolytic to oxidative metabolism across a spectrum of cell types^32,33^.

Metabolomics^34,35^ is a specialized technique based on mass spectrometry that reports on metabolic intermediates and end products in both glycolysis and the citric acid cycle, including glucose, pyruvate, lactate, citrate, succinate, and ATP, among many others. Metabolomics operates on a complementary length scale to PET/MRI and *in vitro* cellular metabolism analyzers by providing information at the tissue level. Unlike PET/MRI, metabolomics requires the destruction of tissue and therefore does not provide functional information. There exists an opportunity for new metabolic tools to bridge the resolution gap between *in vitro* analysis and whole body imaging, while providing kinetic information to complement metabolomics.

We have developed a novel strategy to image the spatiotemporal relationship between glucose uptake and mitochondrial metabolism in an intact tissue microenvironment using intravital microscopy. This approach enables imaging of the major metabolic axes, glycolysis and oxidative phosphorylation, that underpin important tumor phenomena. Two indicators were leveraged to achieve this; 2-[N-(7-nitrobenz-2-oxa-1, 3-diazol-4-yl) amino]-2-deoxy-D-glucose (2-NBDG) is an indicator of glucose uptake and Tetramethylrhodamine ethyl ester (TMRE) reports on mitochondrial membrane potential. Our group^36,37^ and others^38–40^ have extensively validated 2-NBDG as a glucose analog in cells, window chambers, and ectopic and orthotopic tumor models. TMRE, a rhodamine derivative, has been extensively used *in vitro* ^41–43^. To complement previous *in vitro* efforts, we have recently demonstrated through rigorous validation studies that TMRE reports on mitochondrial membrane potential *in vivo* ^44,45^.

In this study, we first established using a combination of optical microscopy and mass spectrometry that there is neither significant optical crosstalk nor chemical crosstalk between 2-NBDG and TMRE in phantoms, making them well suited for simultaneous imaging. However, TMRE uptake was significantly inhibited by simultaneous injection with 2-NBDG *in vivo*. Further investigation demonstrated that the inhibitory effect was due to 2-NBDG temporarily interfering with normal glucose usage which was verified using positive and negative perturbations with 2-DG and glucose, respectively. A staggered delivery scheme, in which TMRE injection was followed by a 2-NBDG injection after a 10-minute delay, mitigated all cross-talk and permitted near- simultaneous *in vivo* microscopy of 2-NBDG and TMRE at the same tissue site. The staggered delivery strategy was evaluated under both normoxic and hypoxic conditions in normal tissues as well as in a murine breast cancer model. The results were consistent with those expected for independent imaging of 2-NBDG and TMRE. In summary, near-simultaneous imaging of TMRE and 2-NBDG provides the unique capability to measure key metabolic endpoints in high resolution with repeatable, *in situ* tumor imaging.

## Results

### There is neither chemical nor optical crosstalk between 2-NBDG and TMRE

**Figure 1:**
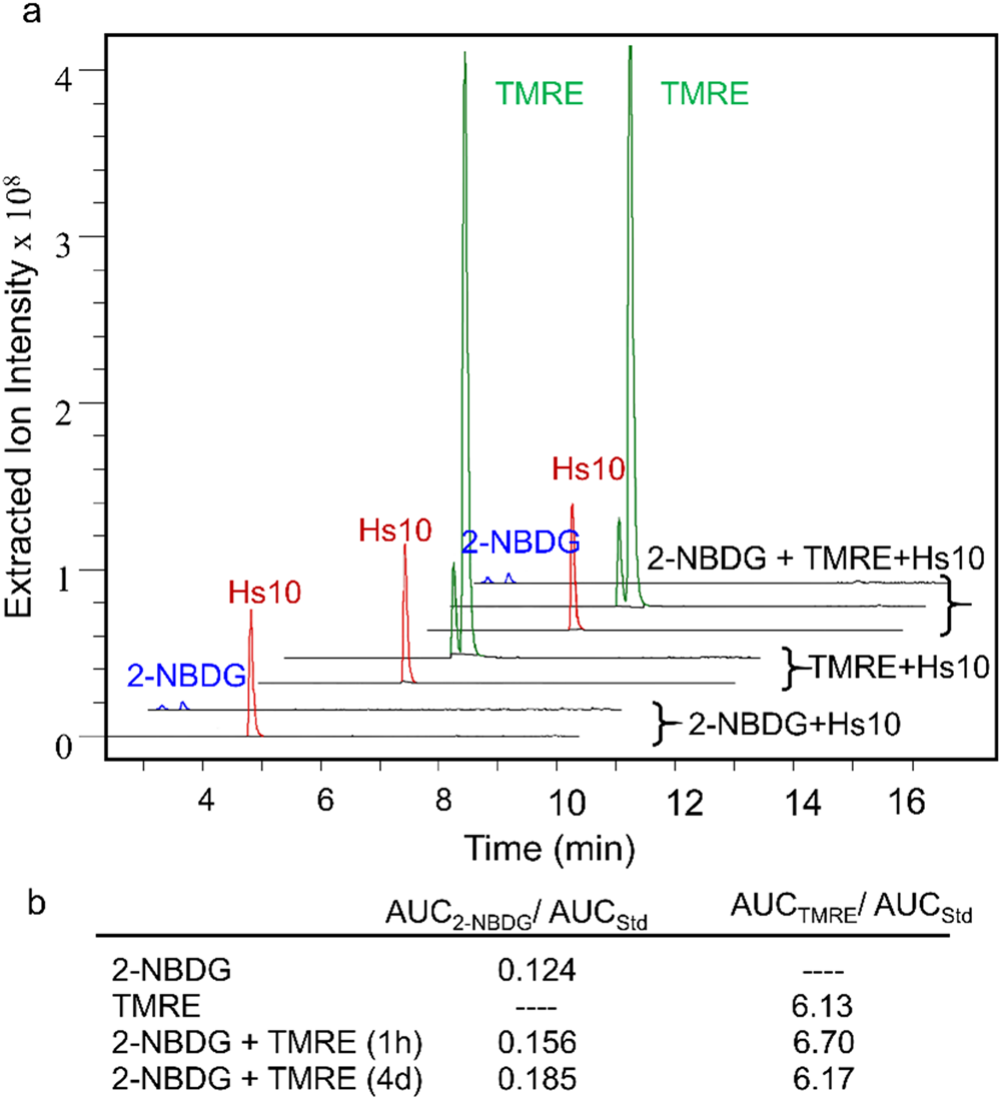
2-NBDG and TMRE are chemically compatible. Solutions containing single-component or combined 2-NBDG and TMRE solutions with an internal standard (Hs10) were analyzed by LC-MS for possible chemical cross-reactivity. (**a**) Extracted ion chromatograms (EIC) showing 2-NBDG as a mixture of alpha- and beta-anomers, TMRE as a mixture of methyl and ethyl esters, and Hs10 as a single peak. Chromatographic features from combined solutions (2-NBDG + TMRE) and single-component solutions (2-NBDG or TMRE alone) were maintained. (**b**) The areas under the curves (AUC) for 2-NBDG (AUC_2-NBDG_), TMRE (AUC_TMRE_), and Hs10 (AUC_Std_) were computed from summation of EI peak integrations related to each compound. Results are shown as the ratio of AUC_TMRE_ or AUC_2-NBDG_ normalized to AUC_Std_.

Liquid chromatography-mass spectrometry with electrospray ionization (ESI-LCMS) analysis of mixed 2-NBDG and TMRE solutions confirmed that there was no inherent chemical reactivity or optical incompatibility between the two fluorophores in the absence of cells or tissue. Figure 1 shows the ESI-LCMS data of four solutions: 1) 100 µM 2-NBDG, 2) 100 µM TMRE, 3) 100 µM 2-NBDG + 100 µM TMRE mixed for 1 hour, and 4) 100 µM 2-NBDG + 100 µM TMRE mixed for 4 days. Each sample contained an internal standard with known spectral features (Hs10) to allow for quantitative analysis. The chromatograms obtained from combined 2-NBDG + TMRE solutions show that all features from the single-component solutions were maintained (Fig. 1a). Further, integration of extracted ion chromatograms revealed that the relative amounts of both 2-NBDG and TMRE, normalized to the internal standard, were not significantly altered after 1 hour or 4 days of mixing (Fig. 1b).

**Figure 2:**
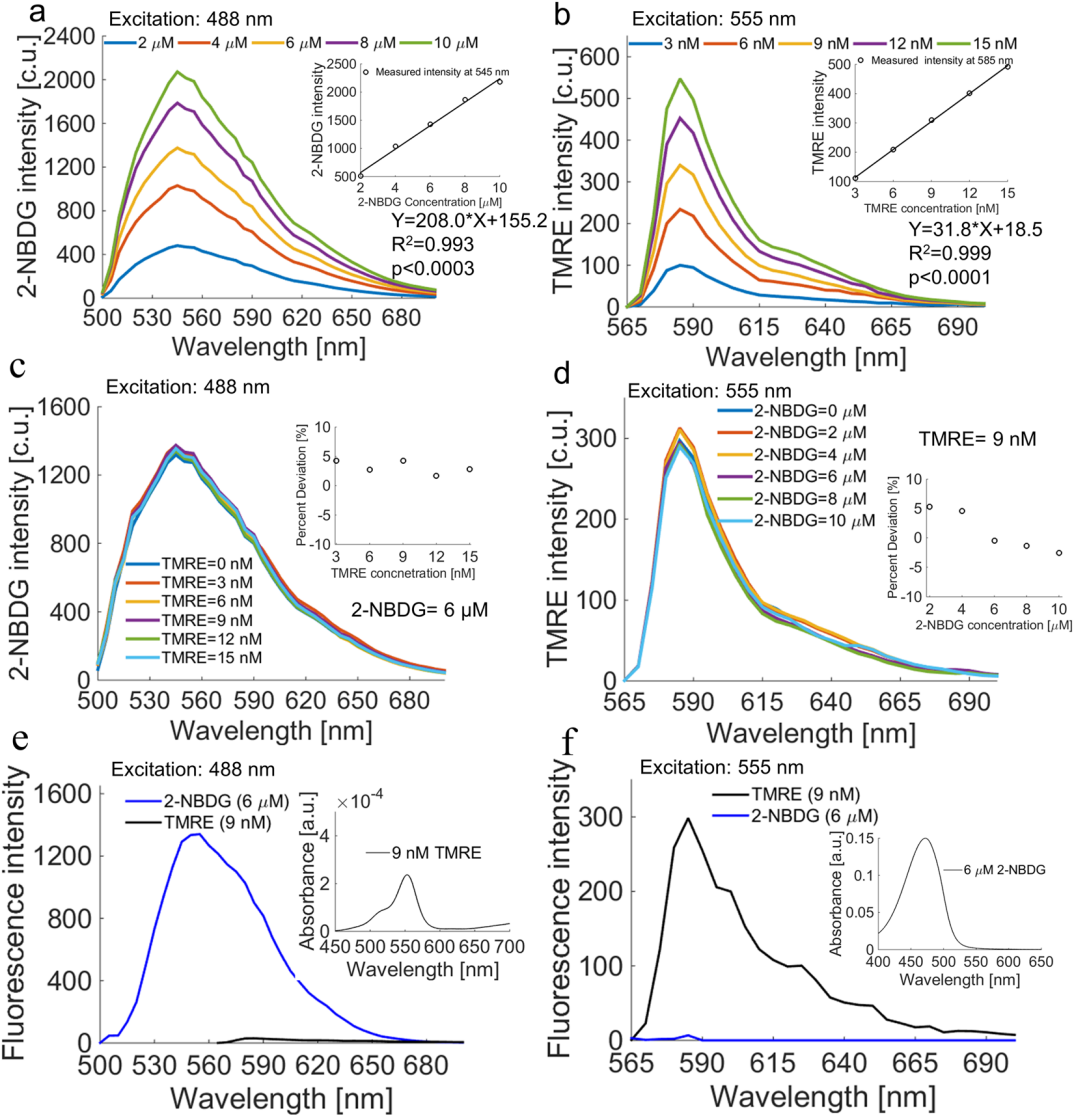
TMRE and 2-NBDG are optically compatible. A set of phantoms containing single-component or combined 2-NBDG and TMRE was tested for optical crosstalk. (**a**) 2-NBDG spectra showed an emission peak of 545 nm and peak intensity increased linearly with concentration. (**b**) TMRE spectra showed an emission peak of 585 nm and peak intensity increased linearly with concentration. (**c**) 2-NBDG intensity was not affected by the presence of TMRE. (**d**) TMRE intensity was not affected by the presence of 2-NBDG. The reduced scattering coefficient in all phantoms was 10 cm^−1^. (**e**) TMRE emits negligible fluorescence compared to 2-NBDG upon excitation with a 488 nm laser, which was typically used for 2-NBDG excitation. TMRE has negligible absorbance at the 2-NBDG emission band. (**f**) 2-NBDG emits negligible fluorescence compared to TMRE upon excitation with a 555 nm laser, which was typically used for TMRE excitation. 2-NBDG has negligible absorbance at the TMRE emission band. Excitation wavelength (488 or 555 nm) is shown on each panel.

Figure 2 shows representative 2-NBDG (Fig. 2a) and TMRE (Fig. 2b) fluorescence spectra obtained from phantom sets with single-component 2-NBDG or TMRE only. The 2-NBDG and TMRE concentrations in each phantom were determined based on our previous hyperspectral imaging studies^36,37,45^ in which we estimated a range of relevant 2-NBDG and TMRE concentrations in tissues (described in the methods). The 2-NBDG concentrations in these phantoms were varied from 0 to 10 µM in 2 µM increments, while the TMRE concentrations in the phantoms were varied from 0 to 15 nM in 3 nM increments. The 2-NBDG and TMRE emission peaks occur at 545 nm and 585 nm, respectively. A linear correlation between fluorescence intensity and fluorophore concentration was observed for each fluorophore as expected (R^2^ = 0.993 and p < 0.003 for 2-NBDG, R^2^ = 0.999 and p < 0.0001 for TMRE). The phantom studies also demonstrate measurable changes in 2-NBDG and TMRE intensity at concentration increments as low as 2 µM for 2-NBDG and 3 nM for TMRE.

In mixed-component phantoms, 2-NBDG fluorescence intensity at a constant 2-NBDG concentration of 6 µM was unaffected by the addition of variable TMRE concentrations between 0 and 15 nM (Fig. 2c). Similarly, TMRE fluorescence intensity at a constant TMRE concentration of 9 nM was minimally affected even when the highest biologically relevant concentration of 2-NBDG (10 µM) was added (Fig. 2d). Figure 2e shows that TMRE emits negligible fluorescence compared to 2-NBDG upon excitation with the 488 nm laser typically used for 2-NBDG excitation. Also, TMRE has negligible absorbance at the 2-NBDG emission band. Figure 2f shows that 2-NBDG emits negligible fluorescence compared to TMRE upon excitation with the 555 nm laser typically used for TMRE excitation. Moreover, 2-NBDG has negligible absorbance at the TMRE emission band. Taken together, the phantom study and ESI-LCMS results indicate that there is neither significant optical nor chemical crosstalk between 2-NBDG and TMRE, showing that they are suitable for combined imaging *in vivo*.

### 2-NBDG uptake is unaffected by simultaneous injection with TMRE, while TMRE uptake is significantly inhibited by simultaneous injection with 2-NBDG

**Figure 3:**
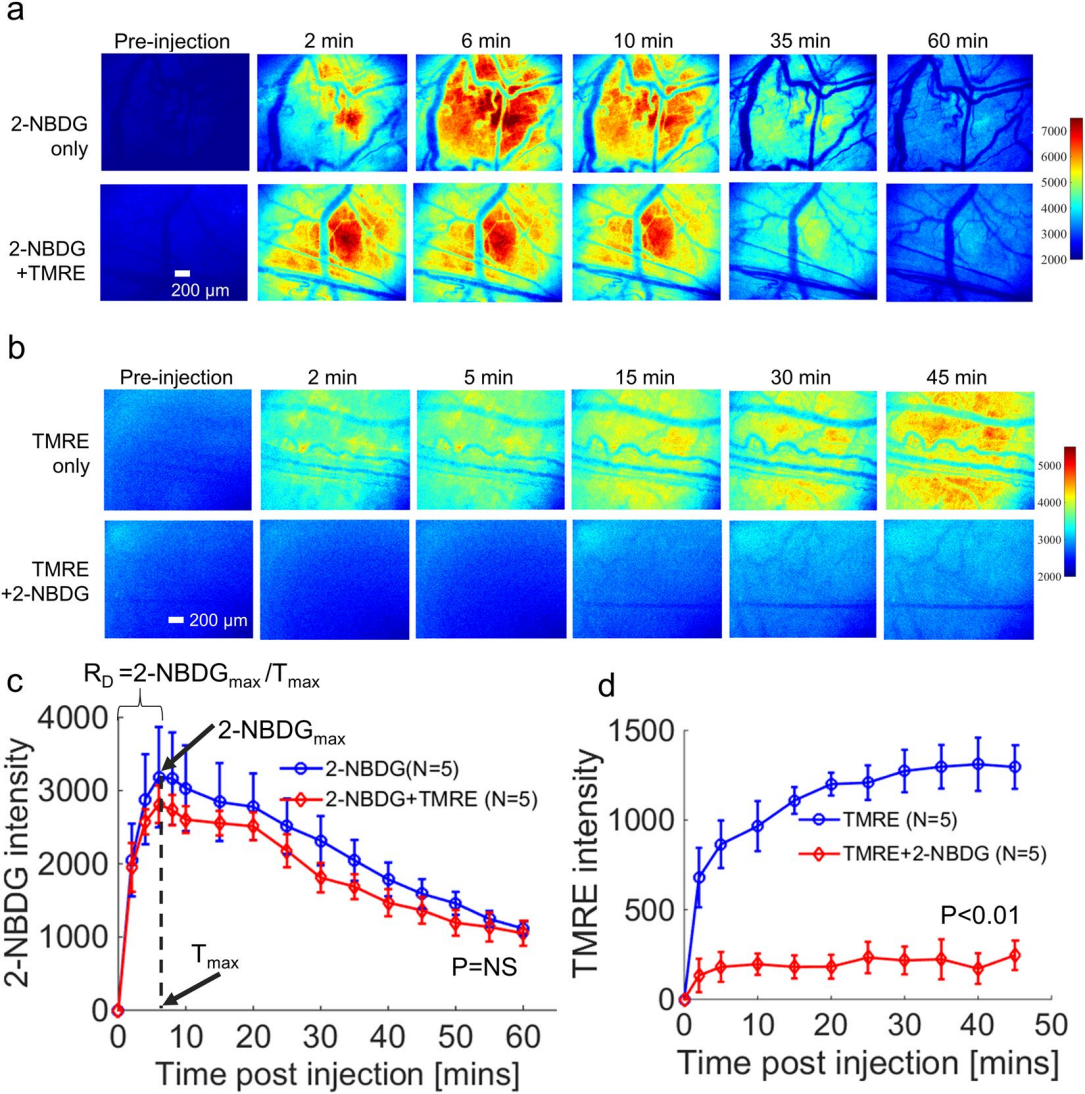
2-NBDG uptake is unchanged by simultaneous injection with TMRE, while TMRE uptake is significantly inhibited by simultaneous injection with 2-NBDG. 2-NBDG and TMRE kinetic imaging was performed on non-tumor murine dorsal window chambers after they received a simultaneous injection (2-NBDG + TMRE) or an independent injection (2-NBDG or TMRE alone). (**a**) Representative 2-NBDG uptake time course images for simultaneous injection and independent 2-NBDG injection. (**b**) Representative TMRE uptake time course images for simultaneous injection and independent TMRE injection. (**c**) Mean uptake kinetics of 2-NBDG. R_D_ refers to the rate of delivery of 2-NBDG. 2-NBDG_max_ = the peak intensity of 2-NBDG. T_max_ = time (in seconds) at which 2-NBDG_max_ occurs. (**d**) Mean uptake kinetics of TMRE. NS = not significant. N = 5 mice/group. Comparison of mean kinetic curves across animal groups was performed with a two-way Analysis of Variance (ANOVA) test using the MATLAB (Mathworks, USA) statistics toolbox.

Figure 3 shows representative results of 2-NBDG or TMRE imaging in animals receiving a simultaneous injection (2-NBDG + TMRE) or an independent injection (2-NBDG or TMRE alone). Figure 3a shows that 2-NBDG fluorescence is negligibly attenuated by the presence of TMRE when both fluorophores are injected simultaneously. Mean kinetic curves in Fig. 3c further demonstrate that the presence of TMRE has negligible effect on the fluorescence of 2-NBDG even when the two fluorophores are injected simultaneously (p = NS for 2-NBDG vs. 2-NBDG + TMRE). The kinetic curves can be used to create a delivery correction factor (R_D_) for 2-NBDG uptake, as demonstrated in subsequent figures. Figure 3b shows that TMRE fluorescence is significantly attenuated by the presence of 2-NBDG when both fluorophores are injected simultaneously. Figure 3d demonstrates that TMRE uptake kinetics are significantly affected by the presence of 2-NBDG when the two probes are injected simultaneously (p < 0.01 for TMRE vs. TMRE + 2-NBDG).

### Attenuation effect of 2-NBDG on TMRE fluorescence is attributed to 2-NBDG interference with normal glucose usage during glycolysis

**Figure 4:**
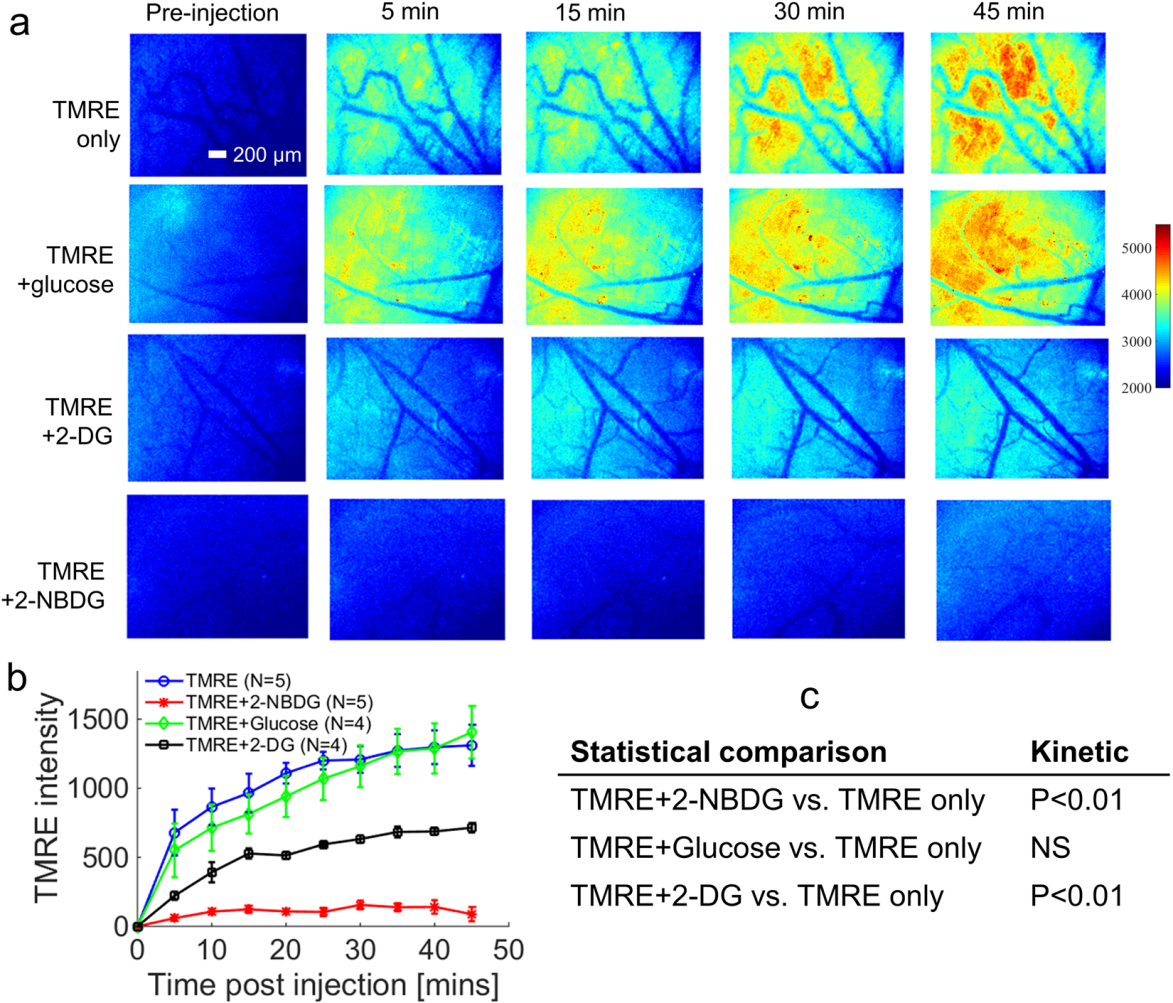
Simultaneous injection with glucose does not affect TMRE uptake, while simultaneous injection with 2-DG decreases TMRE uptake by half. TMRE kinetic imaging was performed on murine dorsal window chambers after they received simultaneous injection of TMRE and glucose, 2-DG, or 2-NBDG. (**a**) Representative TMRE uptake time course images for each group. (**b**) Mean uptake kinetics of TMRE for each group. (**c**) Statistical comparison of the mean kinetic curves for the simultaneous injection groups vs. TMRE alone. NS = not significant. N = 4–5 mice/group. Comparison of mean kinetic curves across animal groups was performed with a two-way Analysis of Variance (ANOVA) test using the MATLAB (Mathworks, USA) statistics toolbox.

We hypothesized that the source of crosstalk was the interference of 2-NBDG with glycolysis. To test this hypothesis, either glucose (normal glycolytic substrate) or 2-DG (shown to inhibit glycolysis^46^) was simultaneously injected with TMRE. Figure 4a shows time course images from animals that were injected with TMRE alone, TMRE and glucose simultaneously, TMRE and 2-DG simultaneously, or TMRE and 2-NBDG simultaneously. Figure 4b demonstrates that TMRE + 2-DG caused altered kinetics compared to TMRE only (p < 0.01 for TMRE vs. TMRE + 2-DG). This was similar to the effect of 2-NBDG (p < 0.01 for TMRE vs. TMRE + 2-NBDG). However, the simultaneous injection of TMRE and glucose had no effect on TMRE kinetics (p = NS for TMRE vs. TMRE + glucose). These results indicate that simultaneous injection with 2-NBDG attenuates the TMRE signal by temporarily interfering with normal glucose usage. This data suggests that staggering the 2-NBDG injection following TMRE injection should enable combined use of the probes.

### A staggered injection strategy enables near-simultaneous microscopy of 2-NBDG and TMRE uptake *in vivo*

**Figure 5:**
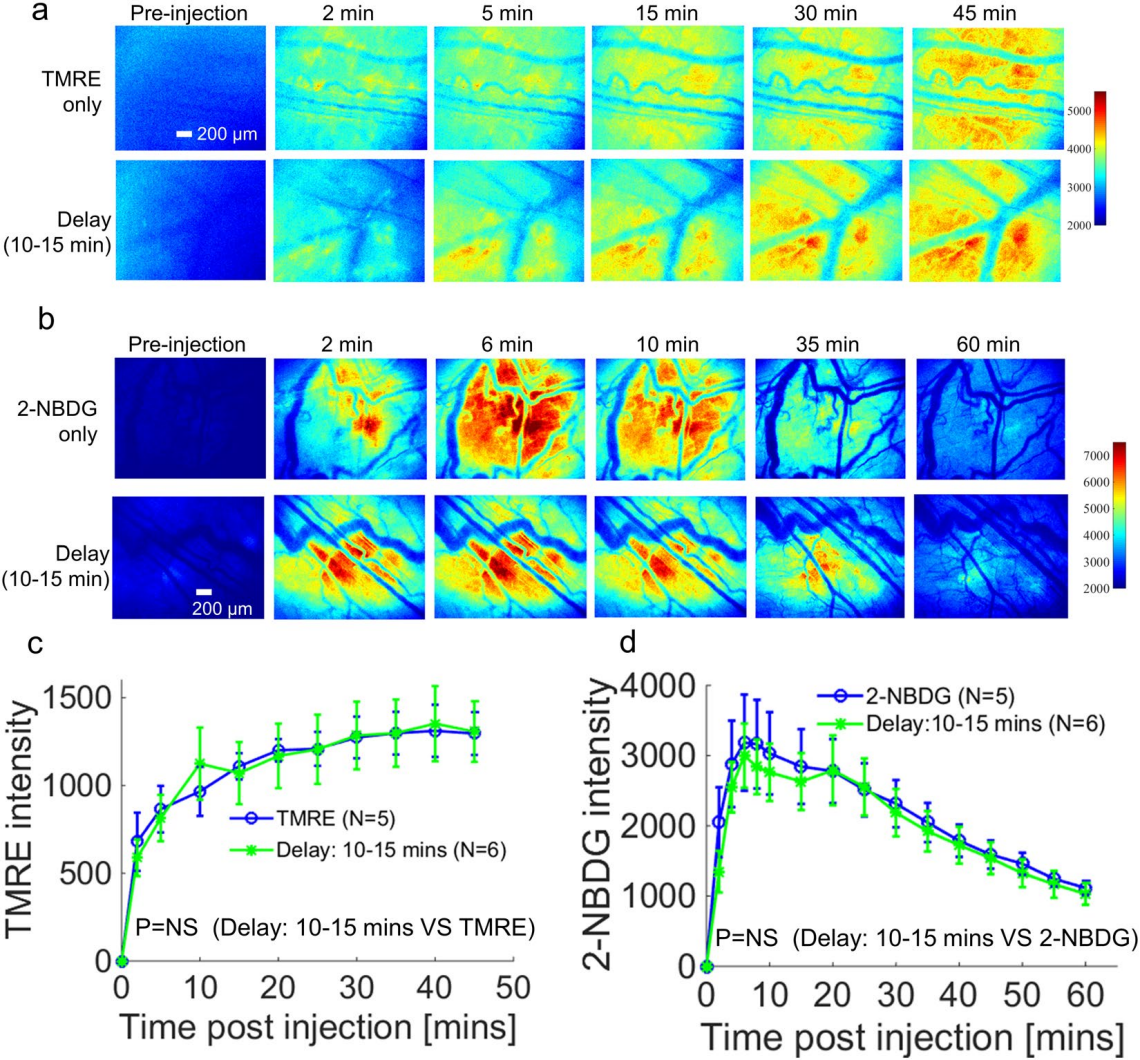
A sequential injection strategy rescues TMRE intensity from the decrease caused by simultaneous injection with 2-NBDG. TMRE and 2-NBDG kinetic imaging was performed on murine dorsal window chambers after they received one of three distinct injection strategies: (1) TMRE alone, (2) 2-NBDG alone, (3) TMRE followed by 2-NBDG with a 10–15 min delay. (**a**) Representative TMRE uptake time course images for the sequential injection strategy and independent TMRE injection. (**b**) Representative 2-NBDG uptake time course images for the sequential injection strategy and independent 2-NBDG injection. (**c**) Mean uptake kinetics of TMRE for each injection strategy. (**d**) Mean uptake kinetics of 2-NBDG for each injection strategy. NS = not significant. N = 5–6 mice/group. Comparison of mean kinetic curves across animal groups was performed with a two-way Analysis of Variance (ANOVA) test using the MATLAB (Mathworks, USA) statistics toolbox.

Figure 5 shows that a sequential injection strategy prevents attenuation of TMRE uptake. TMRE was injected first, followed by 2-NBDG injection after a 10–15 minute delay. When sequential injection with a 10–15 minute delay was used, TMRE fluorescence closely recapitulated the results that were obtained when TMRE was administered alone (Fig. 5a). TMRE time course images from each group shown in Fig. 5a were used to create kinetic curves (Fig. 5c). Figure 5c clearly demonstrates that the sequential injection of TMRE followed by 2-NBDG restored the expected kinetics (p = NS for TMRE vs. Delay: 10–15 min). Figure 5b shows that the fluorescence of 2-NBDG was negligibly affected by the presence of TMRE when TMRE and 2-NBDG were injected sequentially with a 10–15 minute delay. Mean kinetic curves in Fig. 5d further confirm that sequential injection does not affect 2-NBDG kinetics (p = NS for 2-NBDG vs. Delay: 10–15 min).

### Imaging of TMRE and 2-NBDG with a staggered injection strategy captures the expected metabolic responses to hypoxia

**Figure 6:**
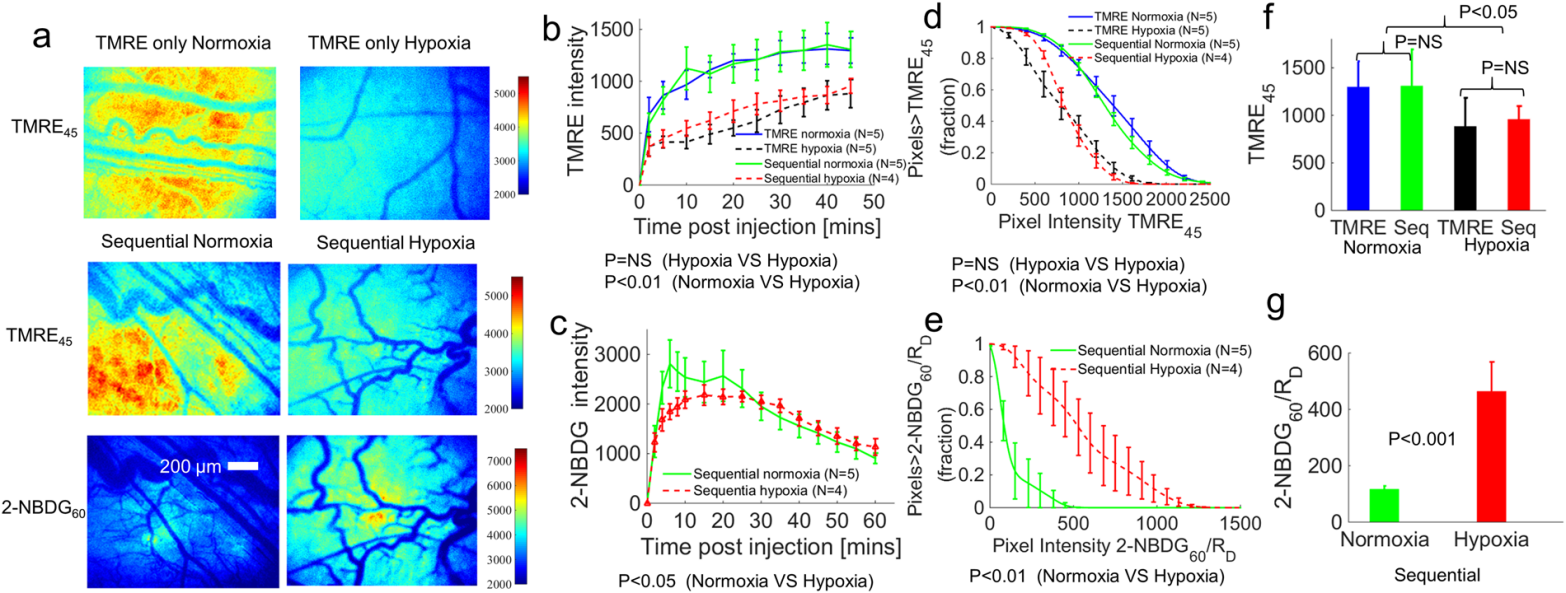
TMRE uptake decreases and 2-NBDG uptake increases in dorsal window chambers under hypoxic gas breathing (10% oxygen). Normal dorsal window chambers were imaged with TMRE (first) and 2-NBDG (second) with a 10–15 min delay between injections. TMRE uptake and 2-NBDG uptake were captured under either normoxia or hypoxia. (**a**) Representative images for each test group. (**b**) Mean uptake kinetics of TMRE. (**c**) Mean uptake kinetics of 2-NBDG. (**d**) Pixel distribution curves show the mean distribution of pixels from TMRE images taken at 45 minutes (TMRE_45_) for each group. (**e**) Pixel distribution curves show the mean distribution of pixels of delivery-corrected 2-NBDG images taken at 60 minutes (2-NBDG_60_/R_D_) for each group. (**f**) Mean intensity from TMRE_45_ images. (**g**) Mean intensity from 2-NBDG_60_/R_D_ images. NS = not significant. N = 4–5 mice/group. Comparison of mean kinetic curves across animal groups was performed with a two-way Analysis of Variance (ANOVA) test using the MATLAB (Mathworks, USA) statistics toolbox. Comparison of mean pixel distribution curves across animal groups was performed with a Kolmogorov-Smirnov (KS) test using the MATLAB (Mathworks, USA). Comparison of the mean intensity of TMRE_45_ or 2-NBDG_60_/R_D_ across animal groups was performed with two sample t-tests using the MATLAB (Mathworks, USA) statistics toolbox.

Figure 6 shows the results of TMRE and 2-NBDG imaging in animals under hypoxic conditions (10% oxygen) that received a TMRE injection only or a sequential injection of both agents with a 10–15 minute delay between the administration of TMRE and 2-NBDG. As shown in Fig. 6a, TMRE intensity at 45 minutes (TMRE_45_) decreased significantly under hypoxia compared to normoxia (21% oxygen) when the animals received either an independent or sequential injection strategy. In contrast, 2-NBDG intensity at 60 minutes (2-NBDG_60_) increased under hypoxia compared to normoxia when the sequential injection strategy was used. Figure 6b and c show the mean uptake kinetics of TMRE and 2-NBDG respectively. Pixel distribution curves were created to illustrate the fraction of pixels in each experimental group that exceeds a given fluorescence intensity value (see Methods for details). Figure 6d shows the pixel distribution curves generated from TMRE images at 45 minutes (TMRE_45_) for both injection strategies and both oxygenation conditions. Figure 6e shows the pixel distribution curves generated from the 2-NBDG images at 60 minutes (2-NBDG_60_) divided by the rate of delivery (R_D_), as shown in Fig. 3c. The individual pixel distribution curves from the animals in each test group were averaged to create the curves shown (mean ± SE). Figure 6f and g show the mean intensity from TMRE_45_ images and mean intensity from 2-NBDG_60_/R_D_ images, respectively. TMRE_45_ decreased significantly during hypoxia (p < 0.05), and 2-NBDG_60_/R_D_ increased significantly during hypoxia (p < 0.001).

### Imaging of TMRE and 2-NBDG with a staggered injection strategy captures a distinct metabolic phenotype in 4T1 tumors relative to non-tumor tissue

**Figure 7:**
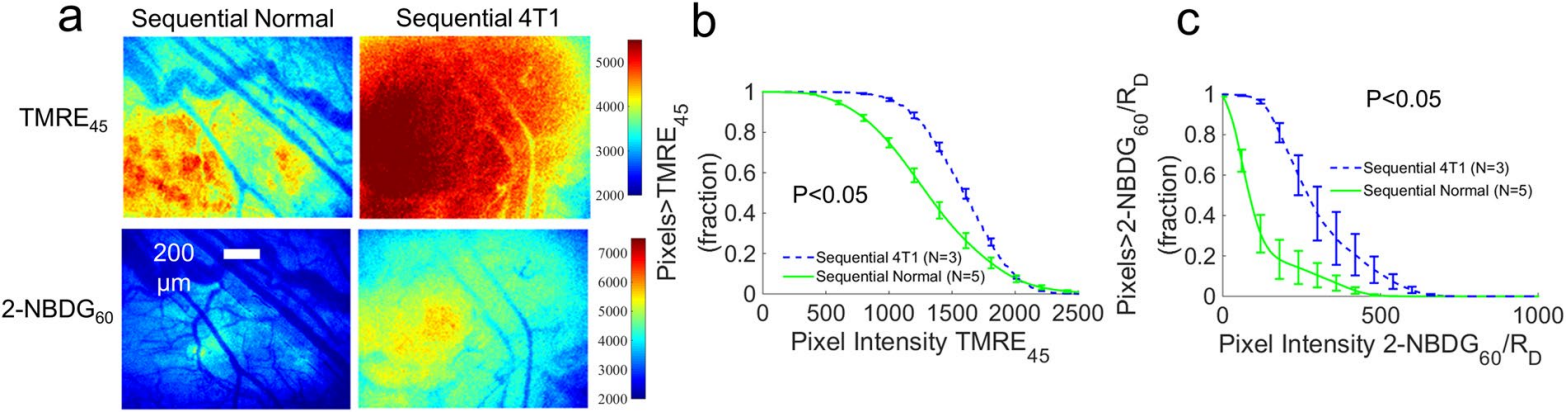
TMRE uptake and 2-NBDG uptake are increased in 4T1 tumors relative to normal tissues. Normal and 4T1 dorsal window chambers were imaged with TMRE (first) and 2-NBDG (second) with a 10–15 min delay between injections. (**a**) Representative images for each test group. (**b**) Pixel distribution curves show the mean distribution of pixels from TMRE images taken at 45 minutes (TMRE_45_) for each group. (**c**) Pixel distribution curves show the mean distribution of pixels of delivery-corrected 2-NBDG images taken at 60 minutes (2-NBDG_60_/R_D_) for each group. NS = not significant. N = 3–5 mice/group. Comparison of mean pixel distribution curves across animal groups was performed with a Kolmogorov-Smirnov (KS) test using the MATLAB (Mathworks, USA).

Figure 7 shows the results of TMRE and 2-NBDG imaging in animals with 4T1 tumors that received a sequential injection of TMRE (first) and 2-NBDG (second) with a 10–15 minute delay. Figure 7a shows TMRE imaging at 45 minutes post TMRE injection and 2-NBDG imaging at 60 minutes post 2-NBDG injection in representative normal and 4T1 tumor-bearing window chambers. Figure 7b and c show the pixel distribution curves generated from images of TMRE_45_ and 2-NBDG_60_/R_D_ respectively (see Methods for details). The individual pixel distribution curves from the animals in each test group were averaged to create the curves shown (mean ± SE). Figure 7b shows that TMRE_45_ increased significantly in 4T1 tumors compared to non-tumor tissues (p < 0.05). Similarly, Fig. 7c shows that 2-NBDG_60_/R_D_ increased significantly in 4T1 tumors compared to normal tissues (p < 0.05). These results are consistent with previous studies that evaluated TMRE and 2-NBDG uptake in 4T1 independently^45^.

## Discussion

Near-simultaneous high-resolution imaging of mitochondrial membrane potential and glucose uptake in living animals is well poised to enable unprecedented studies of metabolism in a variety of important disease models and, in particular, cancer. Though several important metabolic imaging techniques are already being used^20^, our complementary fluorescence-based technique can be coupled with a variety of optical technologies to provide a resolution that enables investigation of the spatiotemporal relationship between glycolysis and oxidative phosphorylation at the tissue microenvironment level^47^. The technique can be utilized to assess novel therapies targeted at tumor metabolism or to identify the metabolic changes that mark response or resistance in specific cell populations. Further, studies of metabolic symbiosis between tumors and their microenvironments^15^, as well as metabolic responses to environmental stress^48,49^, will benefit from high-resolution, metabolic imaging of the intact tissue microenvironment.

The ability of our microscope to image superficial tissues at capillary-level resolution with a millimeter-scale single frame field of view makes it particularly useful for imaging the tissue microenvironment in window chamber models. Dorsal window chambers are by design optically thin and therefore provide an excellent model system to image via microscopy^50^, where there is an inherent tradeoff between sensing depth and lateral resolution. The existing window chamber imaging techniques either provide a large field of view (wide-field imaging systems^36,51^) or high resolution (multiphoton^52^ or confocal microscopes^53^), but not necessarily both. Our microscope has both high resolution (~2.2 µm) and a millimeter-scale single frame field of view (2.1 mm × 1.6 mm), which makes it well-suited to image normal tissue and small tumors in a window chamber model. Further, it has a sensing depth of approximately 500 µm at the wavelength band for fluorescence imaging (i.e. 545 nm for 2-NBDG fluorescence and 585 nm for TMRE fluorescence)^54^. The phantom studies showed measurable changes in 2-NBDG and TMRE intensity when the concentration was varied as little as 2 µM for 2-NBDG and 3 nM for TMRE.

The stable binding and rapid equilibration of TMRE enabled us to perform simultaneous imaging of mitochondrial membrane potential and glucose uptake by injecting TMRE first followed with 2-NBDG injection after a delay. It should be noted that there are several fluorescent mitochondrial dyes that can be used for MMP measurements^43^. Different probes are recommended for each usage paradigm, depending on the probe’s uptake kinetics, concentration, and mitochondrial binding affinity. Rhodamine 123 is recommended for applications that seek to measure rapid changes in membrane potential^43^. JC-1 dye^55^ is commonly used for measurement of transient, non-stable changes in MMP. Both TMRE and TMRM (Tetramethylrhodamine, methyl ester)^43^ are recommended for measurement of pre-existing differences in MMP, such as the stable differences between tumor groups and normal tissue that we desire to observe. TMRM can be used when only a short binding period is needed to minimize disturbance to electron transport^43,56^. However, our primary goal in this study was to determine appropriate time points for combined imaging with 2-NBDG, which has its own unique delivery and uptake kinetics. We thus chose to use TMRE because of its fast equilibration and stable binding, which maximized the likelihood of finding a time point that was appropriate for imaging both probes. To characterize TMRE’s basic *in vivo* properties, our study expands upon previous work by including a recommended TMRE dose, providing TMRE uptake kinetics in both normal and tumor models, and validating TMRE imaging through multiple perturbations.

Previous studies have seen a range of interactions that can occur when multiple compounds are given simultaneously^57–59^, thus changing their kinetics or preventing full accumulation of the compounds. We established in our tissue mimicking phantom study that no detectable chemical nor optical interaction was seen between the fluorophores. Consistent with phantom studies, the tissue studies demonstrated that TMRE negligibly affected the fluorescence of 2-NBDG. Since the presence of TMRE did not affect 2-NBDG uptake, this experiment also importantly confirmed that low concentrations of TMRE reach the tissue, and TMRE thus operates in the non-quenching range. As a result, TMRE fluorescence can be interpreted as dye accumulation corresponding to more polarized mitochondria^60^.

We observed that simultaneous injection of 2-NBDG and TMRE changed TMRE uptake. To understand the inhibitory mechanism that 2-NBDG exerts on TMRE, we imaged TMRE during co-injection with either glucose or 2-DG. Co-injection with glucose had no effect on the TMRE signal; however, co-injection with 2-DG caused a decrease in TMRE uptake similar to that caused by 2-NBDG. Glucose, 2-DG, and 2-NBDG are all taken up by GLUT transporters and phosphorylated by hexokinase^33,61–63^. However, only glucose continues fully through glycolysis to pyruvate, which is converted to acetyl-CoA and fed into the TCA cycle^64^. 2-NBDG and 2-DG remain trapped in the cytoplasm after phosphorylation^62,63^, which has been shown to have an inhibitory effect on glycolysis. The resulting decrease in pyruvate to the TCA cycle may therefore be responsible for a drop in mitochondrial membrane potential and TMRE uptake. We know that, at the concentration used, any metabolic effects of 2-NBDG occur on a short time-scale, since we previously showed that multiple days of 2-NBDG imaging did not cause an order effect^65,66^. It is yet unclear why 2-NBDG caused greater inhibition of TMRE uptake than 2-DG. Compared to other fluorescent glucose probes, 2-NBDG has a low molecular weight (MW = 342) and it directly competes with both glucose and 2-DG for cellular uptake^62,67^. However, 2-DG has an even lower molecular weight (MW = 164) and we hypothesize that this allows it to clear from tissue rapidly. This 2-DG clearance may be responsible for partially restoring TMRE uptake to a level between the 2-NBDG group and the control group.

Toward our ultimate goal of metabolic imaging in diverse cancer applications, our current work in normal tissues served to optimize and validate the sequential injection protocol that enabled near-simultaneous imaging of 2-NBDG and TMRE. Sequential injection of TMRE followed by 2-NBDG with a 10–15 minute delay restored the expected uptake and kinetics of both fluorophores by allowing TMRE to equilibrate in the tissue and bind stably to mitochondria^43^ prior to 2-NBDG injection. Near-simultaneous imaging with the delayed injection strategy consistently yielded results in line with the known metabolic response to hypoxia: increased glucose uptake and decreased mitochondrial metabolism in normal tissue^68^. Imaging in 4T1 window chambers also indicated that the sequential injection strategy developed here was appropriate for small tumors (~6 mm diameter). We saw that 4T1 tumors maintained both increased 2-NBDG uptake and increased TMRE uptake relative to normal tissue, consistent with the findings from our former study^45^ in which 2-NBDG and TMRE were injected in separate cohorts of animals. The average intensity of TMRE_45_ increased ~1.5 fold and the average value of 2-NBDG_60_/R_D_ increased ~3.5 fold in 4T1 tumors compared to normal tissue, which is comparable to the changes observed as a result of hypoxic stress in non-tumor window chambers. While the hypoxia and tumor studies illustrate the dynamic range of TMRE and 2-NBDG imaging, the phantom studies speak to the sensitivity of the technique.

It is interesting to observe that fluorescence signal was not uniform throughout the field of view in the dorsal window chamber studies. There may be multiple biological phenomena that underlie the variable fluorescence signal. Most importantly, our previous studies have shown that vascular oxygenation is spatially heterogeneous, even in non-tumor tissue^36,37,45^. The relationship between oxygenation and metabolism, as demonstrated here by our hypoxic perturbation study, likely influences the regional uptake of both probes. Oxygenation can have profound effects on metabolism; specifically, hypoxia is strongly associated with a shift toward a glycolytic phenotype in normal tissue and particularly in tumors^69^. As tumors grow, they develop natural regions of hypoxia due to the combination of increased oxygen consumption during mitochondrial metabolism^69^, cell growth beyond the oxygen diffusion limit, and impeded delivery due to the immature and tortuous vessels created by angiogenesis^65,70,71^. Our previous work^45^ in which 2-NBDG and TMRE were injected in separate cohorts of animals has demonstrated that decreasing the inspired oxygen concentration to 10% caused profound metabolic effects in a panel of tumor lines (4T1, 4T07 and 67NR). Glucose uptake typically increased when inspired oxygen concentration was decreased to 10%, while TMRE uptake typically decreased under the same forced hypoxic conditions. However, both glucose uptake and TMRE increased during hypoxia in the metastatic 4T1 line. As tumors progress and develop regions of hypoxia, they will be likely characterized by a shift toward increased glucose uptake and decreased MMP, though highly aggressive tumors may reveal special adaptations to hypoxic stress.

High-resolution imaging of glycolytic and mitochondrial endpoints will prove useful to study not only cancer, but also diabetes and other diseases characterized by metabolic aberrations, which until now have suffered from the lack of repeatable, high resolution metabolic imaging technologies. The extensive use of *in vitro* cellular metabolism assays in the fields of immunology, neurobiology, nutrition, and cardiovascular research, among many others, highlights the widespread usefulness of metabolic measurement technologies. By enabling *in vivo* studies of glucose uptake and mitochondrial membrane potential at a length scale and resolution that complement existing methods, our imaging technique has the potential to fill an important need and facilitate novel transdisciplinary studies of metabolism.

## Methods

### Liquid chromatography-mass spectrometry of fluorophore samples

Quantitative LCMS was performed on samples of 2-NBDG and TMRE with an internal standard, 2-(((1 R,4 R)-4-Hydroxycyclohexyl)amino)-4-(3,6,6-trimethyl-4-oxo-4,5,6,7-tetrahydro-1H-indazol-1-yl)benzamide (Hs10), prepared as previously described^72^, to analyze for fluorophore stability. Four methanolic solutions were prepared: (i) 100 µM 2-NBDG, 100 µM Hs10; (ii) 100 µM TMRE, 100 µM Hs10; (iii) 100 µM 2-NBDG, 100 µM TMRE, 100 µM Hs10 (incubated 1 hour at 25 °C), and (iv) 100 µM 2-NBDG, 100 µM TMRE, 100 µM Hs10 (incubated 4 days at 25 °C). Electrospray Ionization (ESI) LCMS analysis was performed using an Agilent 1200 Series liquid chromatography unit with Agilent Ion Trap 6310 mass spectrometer detection (Agilent Technologies, Santa Clara, USA). Chromatography was performed on an Agilent Eclipse Plus C18 column, 5 µm, 4.6 × 150 mm, 10 µL injection volume, using solvents A: 0.2% formic acid in water; B: 0.2% formic acid in acetonitrile; gradient separation method: 0–100% B over 9 minutes, flow rate 1 mL/min. Extracted Ion Chromatograms (EIC) were created by extraction of m/z signals for all ions related to analytes (2-NBDG, TMRE) and Hs10 from total ion chromatograms. EI for 2-NBDG: m/z [M + H]+ = 343.0; [M + Na]+ = 365; [2 M + Na]+ = 707.0. EI for TMRE: m/z [M]+ = 415.0 (ethyl ester); [M]+ = 401.0 (methyl ester). EI for Hs10: m/z [M + H]+ = 411.0. All EI peaks related to analytes and Hs10 were manually integrated. The summation of area under the curve for each analyte (AUC_analyte_) was compared to the AUC for Hs10 from each sample (AUC_Std_). Ratios of AUC_analyte_/AUC_Std_ were used to determine changes in analyte concentration within samples (iii) and (iv) relative to samples (i) and (ii).

### Spectral fluorescence microscopy system

**Figure 8:**
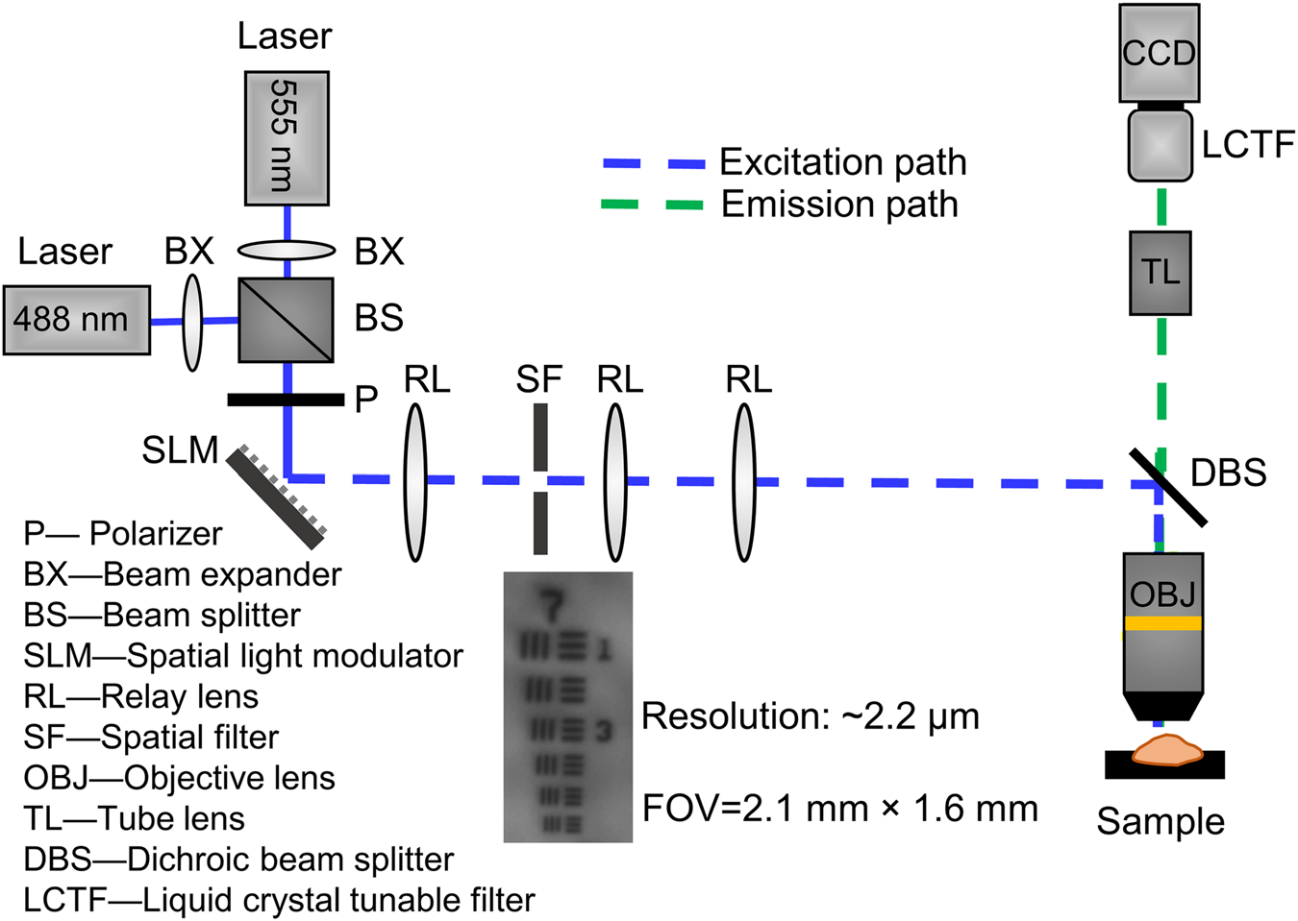
Schematic of the fluorescence spectral imaging system with millimeter-scale field of view and micron-level resolution. The 488 nm laser was used for 2-NBDG imaging while the 555 nm laser was used for TMRE imaging. BX: Beam expander; BS: Beam splitter; CCD: Charge-coupled device; DBS: Dichroic beam splitter; LCTF: Liquid crystal tunable filter; OBJ: Objective lens; P: Polarizer; RL: Relay lens; SF: Spatial filter; SLM: Spatial light modulator; TL: Tube lens.

To further determine whether 2-NBDG and TMRE were suitable for combined imaging, we performed a tissue-mimicking phantom study and animal imaging using a custom designed microscope. In this study, our previously reported microscope^73^ has been modified as shown in Fig. 8 for optical imaging of both phantoms and *in vivo* animal tissue. In the illumination channel, a 488 nm crystal laser (DL488–100-O, Crystal laser, Reno, NV, USA) and a 555 nm crystal laser (CL555-100-O, Crystal laser, Reno, NV, USA) were utilized to excite 2-NBDG and TMRE, respectively. A 505 nm longpass dichroic mirror (DMLP505R, Thorlab, USA) and a 573 nm longpass dichroic mirror (FF573-Di01-25 × 36, Semrock, Rochester, New York, USA) were placed in the beam splitter wheel for 2-NBDG and TMRE imaging, respectively. The key advantage of the fluorescence system is its spectral capability, which is achieved by using a liquid crystal tunable filter (LCTF) (VariSpec VIS-7-35, PerkinElmer, Inc. Waltham, MA, USA) and a high resolution dual-modal charge-coupled device (CCD) (ORCA-Flash4.0, Hamamatsu, Japan). The spectral microscope system was calibrated wavelength by wavelength using a standard lamp source (OL 220 M, S/N: M-1048, Optronic Laboratories, USA).

A Nikon CFI E Plan Achromat 4x objective (NA = 0.1, Nikon Instruments Inc., USA) was used for all imaging studies. The single frame field of view (FOV) and lateral resolution were measured using a 1951 USAF resolution target. The smallest element on the target (group 7, element 6), corresponding to a lateral resolution of 2.2 µm, was clearly resolved as shown in Fig. 8. The single frame FOV was measured to be 2.1 mm × 1.6 mm, and was limited by the illumination beam size rather than the CCD itself. The entire system was controlled via custom-designed LabVIEW software allowing the spectral imaging to be performed automatically and rapidly. Note that while our microscope is capable of optical sectioning due to its structured illumination modality, we did not utilize this feature in the current study.

### Tissue-mimicking phantoms

**Figure 9:**
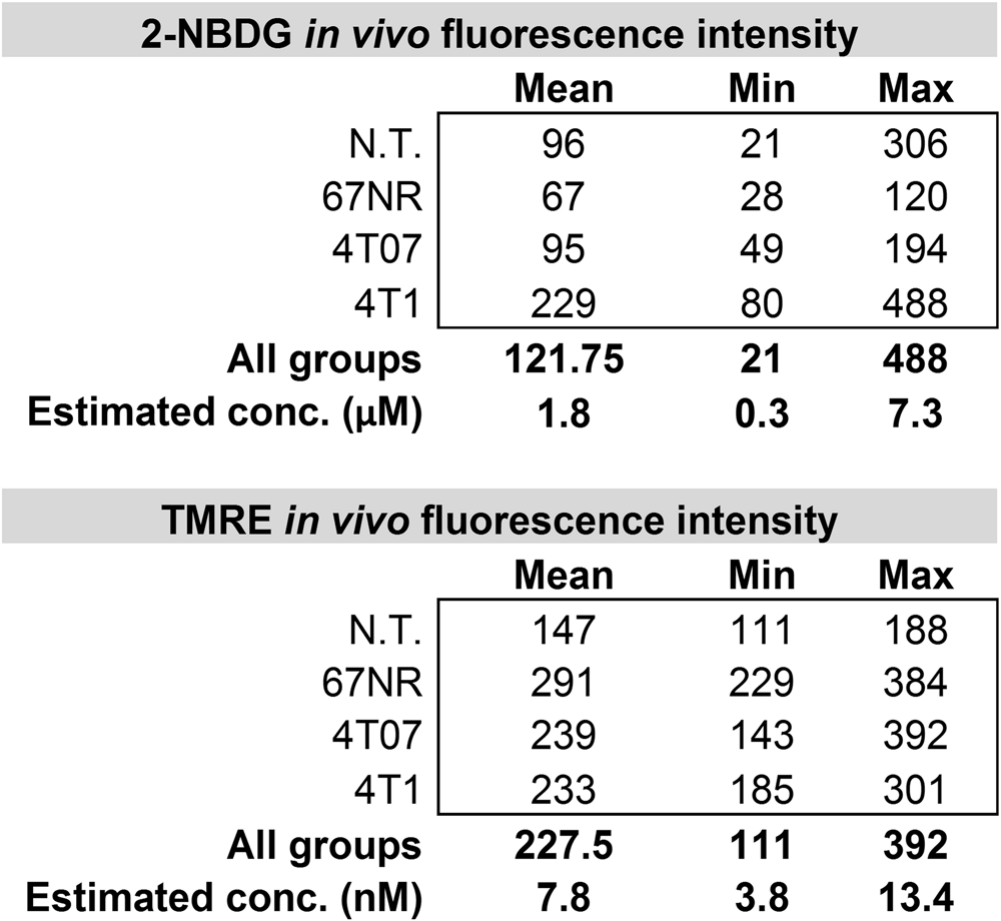
Estimated *in vivo* concentrations of 2-NBDG and TMRE in normal and tumor window chambers using a hyperspectral imaging system^44,45^. Fluorescence images were captured in non-tumor (N.T.) window chambers and in 67NR, 4T07, and 4T1 murine tumors after single injection with 2-NBDG (0.1 mL of 6 mM) or TMRE (0.1 mL of 25 µM). The estimated 2-NBDG and TMRE concentrations were then calculated by comparing *in vivo* fluorescence intensities to fluorescence intensities of tissue-mimicking phantoms imaged with the same instrument settings. Numbers in the tables correspond to fluorescence intensities which were then converted to estimated tissue-level concentrations.

A series of tissue-mimicking phantoms containing 2-NBDG or TMRE at various biologically-relevant concentrations was prepared to validate the system’s spectral capability. The 2-NBDG and TMRE concentrations in each phantom were determined based on our previous hyperspectral imaging study from which we estimated a range of relevant 2-NBDG and TMRE concentrations in animal tissue (Fig. 9). Specifically, the 2-NBDG concentrations in these phantoms were varied from 0 to 10 µM in 2 µM increments, while the TMRE concentrations in the phantoms were varied from 0 to 15 nM in 3 nM increments. Two sets of mixed-component fluorescence phantoms containing both 2-NBDG and TMRE were prepared to investigate potential optical cross-talk between the two fluorophores. In one set of mixed-component phantoms, 2-NBDG concentration was fixed to be 6 µM while TMRE concentrations were varied from 0 and 15 nM. In the second set of mixed-component phantoms, the TMRE concentration was fixed to be 9 nM while 2-NBDG concentrations were varied from 0 to 10 µM. Polystyrene spheres (07310, Polysciences, Warrington, Pennsylvania) were used as the scatterer in all phantoms. The reduced scattering level for all fluorescence phantoms was 10 cm^−1^, which closely mimics the scattering level of window chamber tissue described in literature^74,75^. No absorbers were added to the fluorescence phantoms since the absorption of window chamber tissue is negligible based on previously published reports^74,75^. Deionized water was used to suspend the scattering beads and the fluorophores in each liquid fluorescence phantom. The 2-NBDG (emission peak around 545 nm) fluorescence images were captured automatically from 500 nm to 700 nm in 5 nm increments with the help of the LCTF. In contrast, the TMRE (emission peak around 585 nm) fluorescence images were acquired from 565 nm to 700 nm in 5 nm increments. The integration time for both 2-NBDG and TMRE imaging was set to 1 s for all phantom studies. The absorbance spectra of pure TMRE solution (9 nM) and 2-NBDG solution (6 µM) were measured by a UV-Vis spectrophotometer (Agilent Cary). In all of the fluorescence measurements, background images of phantoms without fluorophores were subtracted from the fluorescence images during data processing.

### Murine dorsal skin flap window chamber model and imaging protocol

All experiments described here were performed in accordance with approved guidelines and regulations. The protocol A114-15-04 was approved by the Duke University Institutional Animal Care and Use Committee (IACUC). We surgically implanted titanium window chambers on the backs of female athymic nude mice (nu/nu, NCI, Frederick, Maryland) under anesthesia (i.p. administration of ketamine (100 mg/kg) and xylazine (10 mg/kg)) using an established procedure^50^. All animals were housed in an on-site housing facility with ad libitum access to food and water and standard 12-hour light/dark cycles. Mice were fasted for 6 hours before imaging to minimize variance in metabolic demand^76^. The animals were randomly assigned to one of the imaging groups listed in Table 1. The fluorescence probes were injected into mice via tail vein. The injection volume was held constant at 100 µL for all experiments. Around 3 to 5 animals were used in each group as specified in the corresponding figures. Imaging groups of normal animals under normoxia were designed to identify the potential biological cross-talk and optimize the protocol for simultaneous imaging of TMRE and 2-NBDG in animals. Imaging groups of normal animals under hypoxia were designed to further validate that the optimized imaging protocol could enable optical measurement of expected responses to known biological perturbations. Imaging groups of 4T1 tumors were designed to test the feasibility of the optimized protocol for cancer metabolic imaging.

**Table 1:** Animal imaging protocols.

Injection protocol	Time point and dosage of tail vein injection
**Normoxia in normal animals**	
TMRE only	t = 0 min: 100 µL of 75 µM TMRE
2-NBDG only	t = 0 min: 100 µL of 6 mM 2-NBDG
TMRE + 2-NBDG	t = 0 min: 100 µL of 75 µM TMRE + 6 mM 2-NBDG
TMRE + Glucose	t = 0 min: 100 µL of 75 µM TMRE + 6 mM glucose
TMRE + 2-DG	t = 0 min: 100 µL of 75 µM TMRE + 6 mM 2-DG
TMRE → 2-NBDG (Delay: 10–15 mins)	t = 0 min: 100 µL of 75 µM TMRE; t = 10–15 min: 100 µL of 6 mM 2-NBDG
**Hypoxia in normal animals**	
TMRE only	t = 0 min: 100 µL of 75 µM TMRE
2-NBDG only	t = 0 min: 100 µL of 6 mM 2-NBDG
Optimal strategy	Optimal strategy determined from normoxic imaging groups
**4T1 tumors**	
Optimal strategy	Optimal strategy determined from normoxic imaging groups

Background fluorescence images of the window chamber were taken prior to the injection of any fluorophores. All of the injections were performed following the protocols listed in Table 1. TMRE fluorescence imaging was performed for 45 minutes with a frequency of every 5 minutes. 2-NBDG imaging was performed for 60 total minutes with a frequency as follows: every 2 minutes for the first 10 minutes and then every 5 minutes for next 50 minutes of imaging. Only TMRE imaging was performed for imaging groups which involved injection of glucose or 2-DG, with the same image capture frequency used in standard TMRE imaging. TMRE imaging was performed at its peak emission wavelength, i.e. 585 nm, while 2-NBDG imaging was performed at 545 nm. The integration time for all *in vivo* fluorescence imaging was set to 5 s. All animals were anesthetized under inhaled isoflurane (1–1.5% v/v) in room air or hypoxic gas during imaging. Each animal was euthanized after the completion of all imaging based on the IACUC protocol.

### Data processing and statistical analysis

Prior to any quantitative image processing, all images from both the phantom study and the animal study underwent background subtraction first and then calibration by a fluorescence slide (DeltaVision, Ex/Em: 488 nm/519 nm), to account for autofluorescence and day-to-day system variation, respectively. Since all of the phantoms were liquid solutions with no identifiable features, it was reasonable to average the spectral images into one spectrum for data analysis for the purpose of demonstrating the spectral capability of the microscopy system. The average intensities of the previously processed fluorescence phantom images at all wavelengths were calculated to form a TMRE fluorescence spectrum or 2-NBDG fluorescence spectrum.

Image processing for the animal data was different compared to the phantom data due to the presence of blood vessels. Previous studies^36^ have revealed that 2-NBDG extravasates into the parenchymal tissue, is taken up by cells, and trapped in the cytosol within a few minutes post tail vein injection. Additional studies^45^ showed that TMRE extravasates into the parenchymal tissue, enters cells, and is localized to mitochondria within 15 minutes post tail vein injection. Minimal fluorescence is observed in large vessels at our imaging time points of 45 minutes (TMRE) and 60 minutes (2-NBDG) because the majority of the dye has already been localized to the cytosol (2-NBDG) or mitochondria (TMRE). Thus, we have excluded these low-signal blood vessel regions during the quantitative analysis in our study to reflect only the 2-NBDG and TMRE uptake in the tissue space. To remove the blood vessels from the quantitative analysis, a manually-traced blood vessel mask was applied to each set of fluorescence images. Only the tissue regions without blood vessels were considered for fluorescence intensity calculations of either TMRE or 2-NBDG. The average intensity values of the non-blood vessel tissue regions at every time point were calculated to generate a time course kinetic curve. Comparison of mean kinetic curves across animal groups was performed using a two-way analysis of variance (ANOVA) test followed by Tukey-Kramer post-hoc tests.

Previous work by our group determined appropriate endpoints for measurement of TMRE and 2-NBDG *in vivo*. We demonstrated that TMRE uptake 45 minutes after injection responded as expected to perturbations of mitochondrial membrane potential in both normal tissue and tumors^45^. TMRE uptake was also robust to minor inter-animal variation in delivery kinetics. On the other hand, we found that although 2-NBDG uptake had reached a stable plateau by 60 minutes after injection (2-NBDG_60_), its final intensity was profoundly influenced by the delivery kinetics of 2-NBDG delivery^36,65^. Accounting for inter-animal differences in 2-NBDG delivery with a correction factor (R_D_ = 2-NBDG_max_/T_max_, as shown in Fig. 3c) resulted in more accurate measurement of glucose uptake following known metabolic perturbations in normal tissue and tumors^36,65^. In the present study, we therefore use the endpoints TMRE_45_ and 2-NBDG_60_/R_D_ to represent TMRE uptake and delivery-corrected 2-NBDG uptake, respectively.

Pixels in the non-vessel space of TMRE_45_ and 2-NBDG_60_/R_D_ images were used to create a pixel distribution curve (1-cumulative distribution) for each animal. The profiles illustrate the fraction of imaged pixels that meet or exceed specific TMRE intensity values or delivery-corrected 2-NBDG intensity values at the time of measurement (t = 45 min, or 60 min, respectively). The individual pixel distribution curves were averaged across multiple animals to create the final curves (mean ± SE). Comparison of TMRE_45_ and 2-NBDG_60_/R_D_ distributions among different imaging groups was performed with a repeated measures Kolmogorov-Smirnov test.

The datasets generated and analyzed during the current study are available from the corresponding author on reasonable request.
